# AUBFM01 Phage as a Therapeutic Candidate Against MDR *Acinetobacter baumannii*: Characterization, and Immune-Aware Profiling

**DOI:** 10.3390/microorganisms14040903

**Published:** 2026-04-16

**Authors:** Dina Kabbara, Layane Nakib, Zahraa Shokor, Tasnime A. Abdo Ahmad, May F. Mrad, Ghassan G. Matar, Esber S. Saba

**Affiliations:** 1 Department of Experimental Pathology, Immunology & Microbiology, Faculty of Medicine, American University of Beirut, Beirut 1107 2020, Lebanon; dmk36@mail.aub.edu (D.K.); ln21@aub.edu.lb (L.N.); zas34@mail.aub.edu (Z.S.); taa53@mail.aub.edu (T.A.A.A.); mm80@aub.edu.lb (M.F.M.); gmatar@aub.edu.lb (G.G.M.); 2 Center of Infectious Diseases Research, Faculty of Medicine, American University of Beirut, Beirut 1107 2020, Lebanon

**Keywords:** *Acinetobacter baumannii*, bacteriophage, multidrug resistance, antibiofilm activity, THP-1 macrophages, immune-aware profiling, *Autographiviridae*

## Abstract

Multidrug-resistant *Acinetobacter baumannii* is a major nosocomial pathogen for which bacteriophages are being explored as alternative antibacterial agents. In this study, we isolated and characterized AUBFM01, a lytic phage active against MDR *A. baumannii*, and performed an initial assessment of its interaction with PMA-differentiated THP-1 macrophages. AUBFM01 was evaluated by host range testing, adsorption and one-step growth assays, lytic activity, stability testing, biofilm disruption, whole-genome sequencing, and flow cytometry-based macrophage profiling. The phage showed rapid adsorption, a short latent period of approximately 30 min, and a burst size of about 165 phage particles per infected cell. It remained stable under moderate temperature and near-neutral pH conditions and significantly reduced preformed *A. baumannii* biofilm biomass in vitro. Genomic analysis identified a 41,354-bp double-stranded DNA genome lacking detectable lysogeny-associated genes, antibiotic resistance determinants, and known bacterial virulence factors. In THP-1 macrophages, AUBFM01 exposure was associated with reduced cell viability and with enrichment of a resting/intermediate-like CD86-defined phenotype among the remaining cells, including after endotoxin reduction. These findings identify AUBFM01 as a lytic anti-Acinetobacter phage with antibiofilm activity and notable macrophage-associated effects that warrant further mechanistic and safety investigation.

## 1. Introduction

*Acinetobacter baumannii* is an important opportunistic pathogen associated with hospital-acquired infections, particularly in critically ill and immunocompromised patients [1,2]. Its remarkable capacity to acquire resistance determinants and persist in the hospital environment has contributed to the global rise of multidrug-resistant (MDR) strains, which are increasingly difficult to treat with conventional antibiotics [3,4]. This therapeutic challenge has renewed interest in bacteriophages as targeted antibacterial agents against MDR pathogens [5,6]. Unlike broad-spectrum antibiotics, phages can selectively infect and lyse susceptible bacterial hosts while potentially sparing the commensal microbiota [7]. In addition, some phages may contribute to biofilm control through mechanisms such as depolymerase-associated degradation of extracellular polymeric substances, although this property is phage dependent [8,9].

Beyond their antibacterial activity, phages are now recognized to interact with mammalian systems in ways that may influence therapeutic performance [7,8]. Increasing evidence suggests that bacteriophages can be internalized by immune cells, affect inflammatory signaling, and alter host responses depending on the phage, the bacterial context, and the degree of preparation purity [9,10]. These interactions may be relevant to both efficacy and safety, particularly in severe infections where bacterial lysis and host inflammatory responses occur simultaneously [11,12]. However, such effects remain incompletely understood and appear to vary substantially between phage preparations [9].

Macrophages are central effectors of innate immunity and play major roles in bacterial clearance, inflammatory amplification, and resolution of infection [13,14]. Because they are likely to encounter therapeutic phages during treatment, macrophages represent a useful model for early immune-aware evaluation of phage preparations [11]. While several studies have reported that phages can influence macrophage behavior, the effects of anti-*Acinetobacter* phages on macrophage-associated phenotypes remain insufficiently characterized [9,12]. Accordingly, the aim of this study was to isolate and biologically characterize a lytic bacteriophage active against multidrug-resistant *Acinetobacter baumannii* and to perform an initial immune-aware assessment of its interaction with PMA-differentiated THP-1 macrophages.

## 2. Materials and Methods

### 2.1. Bacterial Strains and Growth Conditions

*Acinetobacter baumannii* ACN1 served as the primary host strain for phage isolation, propagation, and amplification. Host range was assessed using the double-layer agar method with a panel of nine clinical *Acinetobacter* isolates. All strains were routinely cultured in Luria–Bertani (LB) broth or on LB agar at 37 °C. Liquid cultures were incubated with agitation at 180 rpm unless otherwise indicated. For phage infection experiments, bacterial cells were harvested at mid-exponential phase, corresponding to an optical density at 600 nm (OD_600_) of approximately 0.5.

### 2.2. Phage Isolation, Purification, and Propagation

Phage AUBFM01 was isolated from a mixed sewage sample collected from environmental water sources in Lebanon using the double-layer agar overlay technique with *Acinetobacter baumannii* ACN1 as the host strain. Following successive rounds of plaque purification, the phage was amplified on its host in LB broth at 37 °C with shaking at 180 rpm. After visible lysis, the culture was centrifuged at 10,000× *g* for 10 min at 4 °C to remove bacterial debris, and the supernatant was filtered through a 0.22 µm membrane.

For phage concentration, NaCl was added to the filtered lysate to a final concentration of 1 M and the lysate was incubated on ice for 1 h. After centrifugation at 10,000× *g* for 10 min at 4 °C, polyethylene glycol 8000 was added to the supernatant to a final concentration of 10% (*w*/*v*), followed by incubation overnight at 4 °C. The precipitated phage particles were recovered by centrifugation at 10,000× *g* for 20 min at 4 °C, and the pellet was resuspended in sterile SM buffer (100 mM NaCl, 8 mM MgSO_4_ and 50 mM Tris-HCl. pH 7.5) Phage stocks were stored at 4 °C until further use.

For experiments involving macrophage stimulation, purified phage preparations were further processed using a Pierce™ High-Capacity Endotoxin Removal Spin Column (Thermo Fisher Scientific) according to the manufacturer’s instructions. Endotoxin concentrations were subsequently quantified using the Pierce™ Chromogenic Endotoxin Quant Kit (Thermo Fisher Scientific), and only preparations with endotoxin levels below 0.1 EU/mL were used for immune-cell exposure assays. Infectious phage titers, expressed as plaque-forming units per milliliter (PFU/mL), were determined at each stage using the double-layer agar method.

### 2.3. Phage Biological Characterization

#### 2.3.1. Adsorption Assay

Phage adsorption kinetics were determined by incubating AUBFM01 with exponentially growing *A. baumannii* ACN1 cells at a multiplicity of infection (MOI) of 0.01 in LB broth at 37 °C. Samples were collected every 2 min for 20 min post-infection and immediately centrifuged at 10,000× *g* for 2 min to sediment bacterial cells. The recovered supernatants were titrated by the double-layer agar method to quantify unadsorbed phages. The adsorption rate constant was estimated according to the standard approach described by Kropinski et al., using the time required to reach 50% reduction in unadsorbed phage and the bacterial concentration present during the assay [15].

#### 2.3.2. One-Step Growth Curve

A one-step growth assay was performed to determine the latent period and burst size of AUBFM01. Mid-exponential-phase *A. baumannii* ACN1 cells (OD_600_ ≈ 0.5) were infected with the phage at a multiplicity of infection (MOI) of 0.1 and incubated at 37 °C for 10 min to allow adsorption. The suspension was then centrifuged at 5000× *g* for 5 min to remove unadsorbed phages, and the bacterial pellet was resuspended in pre-warmed LB broth. Samples were collected at 5–10 min intervals over a total period of 60 min, and phage titers were determined immediately using the double-layer agar method. The latent period was defined as the interval between infection and the initial increase in extracellular phage titer, whereas the burst size was calculated as the ratio of the final phage titer to the initial number of infected cells during the exponential rise phase.

#### 2.3.3. Bacteriolytic Activity

The lytic activity of AUBFM01 was assessed using a microplate-based growth inhibition assay. Mid-exponential-phase *A. baumannii* ACN1 culture (OD_600_ ≈ 0.5) was diluted 1:100 in fresh LB broth, and 200 µL aliquots were transferred into 96-well microplates. Phage was added at multiplicities of infection (MOIs) of 10, 1, 0.1, and 0.01. Uninfected bacterial cultures were included as positive growth controls, and sterile LB broth served as a negative control. Plates were incubated at 37 °C with continuous shaking, and OD_600_ measurements were recorded every 30 min over 12.5 h using a microplate reader (Tristar 3). All conditions were tested in triplicate.

#### 2.3.4. Environmental Stability

- **Thermal Stability:** Phage thermal stability was evaluated by incubating lysates (~10^8^ PFU/mL) at temperatures ranging from 10 °C to 70 °C for 60 min in a water bath using a protocol adapted from [16]. Samples were then immediately cooled on ice, and residual infectivity was quantified by the double-layer agar method. Stability was defined as a reduction in phage titer of less than 1 log_10_ compared with the control stored at 4 °C.
- **pH Stability:** Phage pH stability was assessed using a working suspension of approximately 10^8^ PFU/mL, prepared by diluting a concentrated phage stock (~10^10^ PFU/mL) directly into SM buffer previously adjusted to pH values ranging from 2.0 to 13.0. Samples were incubated at 37 °C for 1 h, after which residual viable phage particles were quantified by the double-layer agar method.

### 2.4. Biofilm Disruption Assay

The ability of AUBFM01 to disrupt preformed *A. baumannii* biofilms was evaluated using a crystal violet staining assay. Briefly, biofilms were established in sterile 96-well polystyrene microplates by inoculating each well with 200 µL of a 1:100 dilution of an overnight bacterial culture, followed by static incubation at 37 °C for 24 h. After incubation, planktonic cells were carefully removed, and wells were washed twice with phosphate-buffered saline (137 mM NaCl, 2.7 mM KCl, 10 mM Na_2_HPO_4_, and 1.8 mM KH_2_PO_4_, pH 7.4). Preformed biofilms were then exposed to 200 µL of AUBFM01 (~10^8^ PFU/mL), while wells receiving sterile LB broth served as positive controls, and incubated for an additional 24 h at 37 °C. Following treatment, wells were washed, stained with 0.1% (*w*/*v*) crystal violet for 15 min, and washed again to remove excess stain. The bound crystal violet was solubilized with 95% ethanol, and absorbance was measured at 590 nm (OD_590_). All conditions were tested in five replicates.

### 2.5. Genome Sequencing and Bioinformatic Analysis

Phage genomic libraries were prepared using the Nextera library preparation kit (Illumina, San Diego, CA, USA) and sequenced as paired-end reads (2 × 250 bp) on an Illumina MiSeq platform at the DNA Sequencing Facility of the American University of Beirut. Genome assembly and annotation were performed in a Linux-based command-line environment. Raw sequencing reads were quality filtered and adapter trimmed using Trimmomatic (v0.40) [17]. The processed reads were assembled using SPAdes (v3.15.5) [18], yielding a single contiguous phage genome with an average sequencing depth exceeding 300×. Coding sequences and tRNA genes were predicted and annotated using Pharokka (v1.8.0) [19]. Circular genome maps were generated using PhageScope (v1.3). Proteome-based phylogenetic analysis was performed using ViPTree (v4.0), which infers viral relationships based on whole-genome similarity [20]. The PhageLeads server was used to screen the genome for predicted toxin genes, virulence factors, and antimicrobial resistance determinants [21]. Intergenomic similarity between AUBFM01 and closely related Acinetobacter phages was assessed using VIRIDIC (Virus Intergenomic Distance Calculator) [22].

### 2.6. Macrophage Differentiation and Stimulation

The human monocytic THP-1 cell line (ATCC TIB-202) was cultured in RPMI 1640 medium supplemented with 10% fetal bovine serum (FBS) and 1% penicillin–streptomycin at 37 °C in a humidified 5% CO_2_ atmosphere. THP-1 monocytes were differentiated into macrophage-like cells by exposure to 50 ng/mL phorbol 12-myristate 13-acetate (PMA) for 48 h. After differentiation, cells were washed twice with PBS and allowed to recover in PMA-free medium for 24 h before experimental stimulation.

For immune assays, phage preparations, including AUBFM01 and the unrelated control phages *Klebsiella* phage Miniara127 (PX094878) and *Escherichia* phage AUBRB02 (OY979771), were propagated and concentrated using PEG 8000. Differentiated THP-1 macrophages were seeded at 2 × 10^5^ cells/well and exposed for 24 h to purified phage suspensions at a final concentration of 10^8^ PFU/mL in 200 µL per well, corresponding to approximately 2 × 10^7^ PFU/well. Additional treatment conditions included bacterial lysates prepared from 10^8^ CFU/mL cultures by sonication and subsequent filtration through a 0.22 µm membrane, lipopolysaccharide (LPS; 100 ng/mL) as a positive control, and culture medium alone as an untreated control.

### 2.7. Flow Cytometry and Immunophenotyping

After stimulation, THP-1-derived macrophages were collected and washed with phosphate-buffered saline (PBS). Cell viability was assessed using a fixable viability dye according to the manufacturer’s instructions. Surface staining was performed by incubating cells for 30 min at 4 °C in the dark with fluorochrome-conjugated antibodies against human CD14 (BD Biosciences, 560349), CD11b (BD Biosciences, 564517), CD45 (BD Biosciences, 564105), and CD86 (BioLegend, 305406). After staining, cells were washed to remove excess antibody and fixed in 2% paraformaldehyde prior to acquisition.

Data were acquired on a BD FACSAria flow cytometer, and fluorescence compensation was established using single-stained controls. Debris was excluded based on forward- and side-scatter characteristics, and viable cells were retained for downstream analysis. FCS files were analyzed in R using flowCore (v2.22.0) for preprocessing and Rtsne (v0.17) for dimensionality reduction. t-SNE was used to generate a two-dimensional representation of macrophage phenotypic variation based on the expression of CD14, CD11b, CD45, and CD86 with standard parameter as mentioned in [23]. To identify cell subpopulations in an unsupervised manner, k-means clustering was subsequently applied, and the relative abundance of each cluster was compared across treatment conditions.

### 2.8. Statistical Analysis

All experiments were performed with at least three independent biological replicates unless otherwise indicated. Data are presented as mean ± standard deviation (SD). Statistical analyses were conducted using R (v4.5.3) and RStudio (v2026.01.1). Phage characterization experiments, including adsorption, one-step growth, lytic activity, and environmental stability assays, were primarily performed as descriptive biological characterization assays; accordingly, these data are presented descriptively with SD shown where applicable. For quantitative comparisons between experimental groups involving more than two groups, one-way analysis of variance (ANOVA) followed by Dunnett’s multiple-comparison test. A *p* value of less than 0.05 was considered statistically significant.

## 3. Results

### 3.1. Isolation, Plaque Morphology, and Host Range of Phage AUBFM01

**Figure 1:**
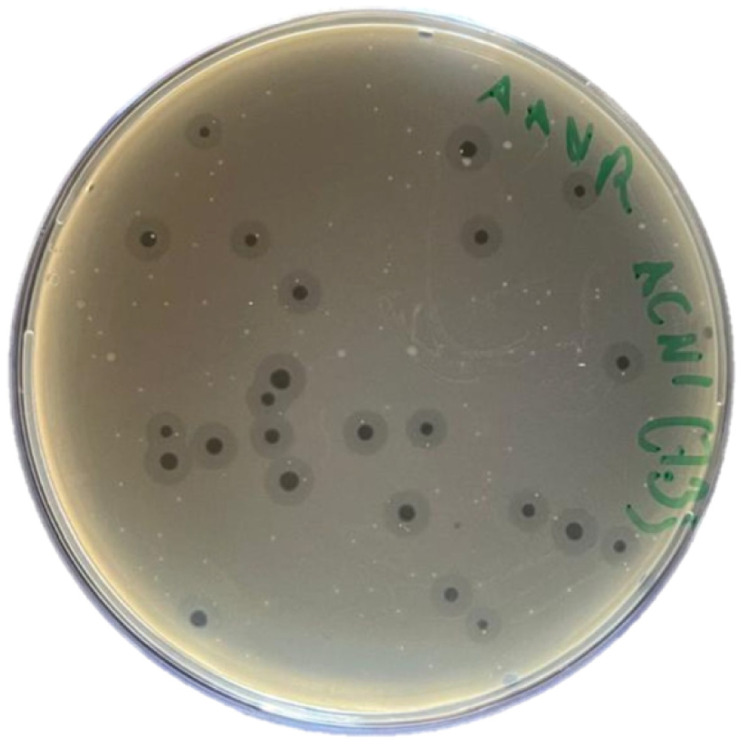
Plaque morphology of phage AUBFM01 on *Acinetobacter baumannii* ACN1 using the double-layer agar assay. AUBFM01 formed clear, circular plaques approximately 2–4 mm in diameter, frequently surrounded by translucent halos suggestive of extracellular matrix or capsular polysaccharide degradation.

Phage AUBFM01 was isolated from an environmental sewage sample using *Acinetobacter baumannii* ACN1 as the enrichment and propagation host. In the double-layer agar assay, AUBFM01 formed clear, circular plaques on ACN1 bacterial lawns, consistent with a lytic infection profile (Figure 1). Plaques measured approximately 2–4 mm in diameter and were often surrounded by translucent halos of variable size. The presence of these halos may suggest polysaccharide-degrading activity, such as depolymerase-associated degradation of surface or extracellular matrix components, although this was not directly confirmed in the present study.

**Figure 2:**
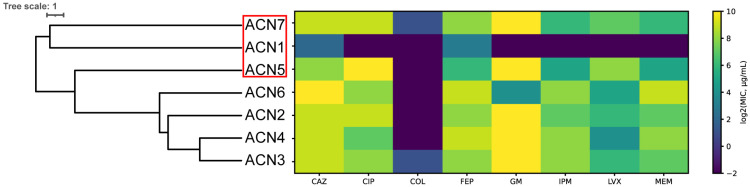
Phylogenetic relationships and antimicrobial susceptibility profiles of the *A. baumannii* clinical isolates used to assess the host range of AUBFM01. The dendrogram shows the relatedness of the tested isolates, and the heatmap displays antimicrobial susceptibility as log_2_MIC (µg/mL). Antibiotics tested were ceftazidime (CAZ), ciprofloxacin (CIP), colistin (COL), cefepime (FEP), gentamicin (GM), imipenem (IPM), levofloxacin (LVX), and meropenem (MEM). The red box highlights the isolates susceptible to AUBFM01 lysis, including the propagation host ACN1 and two additional isolates, ACN5 and ACN7.

The host range of AUBFM01 was evaluated using a panel of clinical *A. baumannii* isolates. To illustrate the diversity of the strain collection, a phylogenetic tree of the bacterial isolates is presented together with their antimicrobial susceptibility profiles (Figure 2). As shown in Figure 2, the tested isolates displayed substantial heterogeneity in their susceptibility patterns, with some strains clustering more closely than others. Within this panel, AUBFM01 exhibited lytic activity against three isolates in addition to the propagation host, indicating that its infectivity was not restricted to ACN1 alone. Although the host range remained limited within the tested collection, the ability of AUBFM01 to infect multiple clinical isolates supports its potential relevance for further evaluation against MDR *A. baumannii* strains. Host range testing in this study was restricted to clinical *A. baumannii* isolates, cross-species lytic activity against non-Acinetobacter pathogens was not assessed.

### 3.2. Phage Adsorption, Replication Kinetics, and Lytic Activity

**Figure 3:**
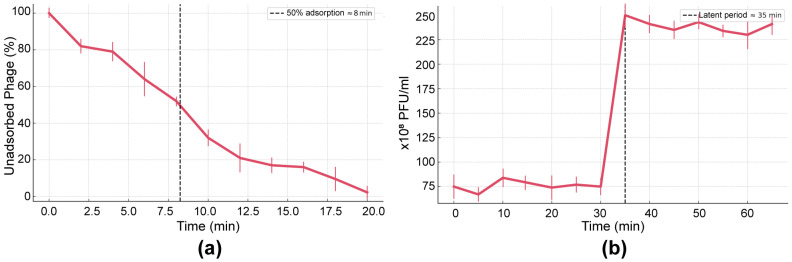
Early infection kinetics of phage AUBFM01 on *A. baumannii* ACN1. (**a**) Adsorption assay showing the progressive decline in unadsorbed phage particles over time. (**b**) One-step growth curve showing an initial latent phase followed by a sharp increase in extracellular phage titer, with phage release beginning at approximately 30–35 min. Together, these data indicate efficient host adsorption, a short latent period, and a burst size of approximately 165 PFU per infected cell. Line connecting points represents means and whiskres represent SD of three biological replicates.

The early infection kinetics of AUBFM01 were evaluated by adsorption and one-step growth assays. In the adsorption assay, the proportion of unadsorbed phage particles decreased progressively over time (Figure 3a). Approximately 80% of the initial phage population remained free in suspension after 4–5 min, decreasing to about 60% by 8 min and to roughly 30% by 12 min. By 15 min, fewer than 20% of phage particles remained unadsorbed, and this value declined to approximately 5% at 20 min, indicating near-complete adsorption to *A. baumannii* ACN1. Based on these data, the adsorption rate constant of AUBFM01 was estimated to be approximately 8.5 × 10^−10^ mL/min, indicating efficient attachment to its host under the tested conditions. Overall, these findings show that AUBFM01 adsorbs efficiently to its host, with most particles becoming cell-associated within the first 15–20 min of contact.

Consistent with this rapid adsorption profile, the one-step growth assay showed a short latent period followed by a marked increase in progeny phage production (Figure 3b). Phage titers remained relatively stable during the initial 30 min, consistent with the latent phase preceding extracellular virion release. A pronounced rise in titer was then observed at approximately 30–35 min, increasing from baseline values of about 70–80 PFU/mL to nearly 250 PFU/mL, after which the curve reached a plateau with only minor variation over the remainder of the experiment. Overall, this growth pattern indicates that AUBFM01 undergoes a relatively rapid intracellular replication cycle with a synchronized release of progeny particles. Based on these data, the latent period was estimated to be approximately 30 min, and the burst size was approximately 165 phage particles per infected cell.

**Figure 4:**
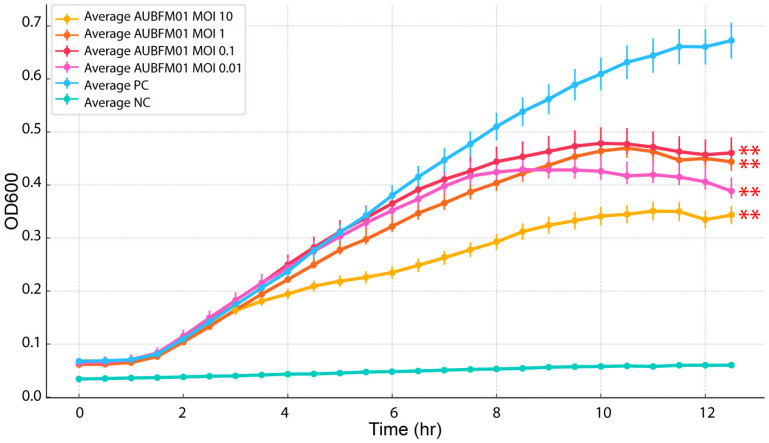
Growth inhibition of *A. baumannii* ACN1 by phage AUBFM01 in liquid culture at different multiplicities of infection (MOIs). Bacterial growth was monitored by OD600 over 12 h in cultures treated with AUBFM01 at MOI 10, 1, 0.1, 0.01, alongside the positive growth control (PC) and negative control (NC). AUBFM01 produced dose-dependent suppression of bacterial growth, with stronger inhibition observed at higher MOIs and partial regrowth at lower phage doses. Line connecting points represents means and whiskers represent SD of three biological replicates; Statistical significance at the final timepoint was assessed relative to the untreated positive control. Significance levels are indicated as follows: ** *p* < 0.01.

The lytic activity of AUBFM01 in liquid culture was further assessed using a microplate-based growth inhibition assay at different input doses (Figure 4). In the untreated bacterial control, optical density increased steadily after the first 2–3 h and reached approximately 0.65–0.68 by 12 h, reflecting robust bacterial growth. In contrast, phage-treated cultures showed dose-dependent growth inhibition. At higher phage doses, bacterial growth was more strongly suppressed, whereas intermediate and lower doses produced only partial inhibition. Under these conditions, bacterial density increased more slowly than in the untreated control and reached lower final OD values, generally plateauing around 0.35–0.47 depending on the phage input. At the lowest doses tested, regrowth became evident at later time points, although optical density remained below that of the untreated culture throughout the experiment. The separation between growth curves became more apparent after approximately 4–6 h, indicating that the antibacterial effect of AUBFM01 was sustained but less complete at lower MOIs. Collectively, these results show that AUBFM01 adsorbs efficiently, replicates rapidly after a short latent period, and exerts dose-dependent lytic activity against *A. baumannii* ACN1.

### 3.3. Environmental Stability of AUBFM01

**Figure 5:**
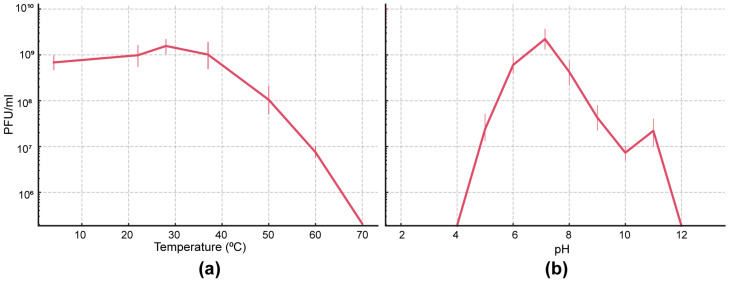
Stability of phage AUBFM01 under different temperature and pH conditions. (**a**) Residual phage titers after exposure to temperatures ranging from 4 to 70 °C show that AUBFM01 remained relatively stable between 4 and 37 °C, with a marked loss of infectivity at higher temperatures. (**b**) Residual phage titers after exposure to pH 2–12 show maximal stability near neutral pH, particularly around pH 7, with substantial reductions in viability under strongly acidic or alkaline conditions. Line connecting points represents means and whiskers represent SD of three biological replicates.

The stability of AUBFM01 under different temperature and pH conditions was evaluated by measuring residual phage titers after exposure to each condition (Figure 5a,b). Overall, AUBFM01 remained stable across a moderate range of environmental conditions but showed a marked reduction in infectivity under more extreme temperature and pH conditions.

Thermal stability analysis showed that AUBFM01 retained substantial infectivity over an intermediate temperature range (Figure 5a). Phage titers remained relatively stable between 4 °C and 37 °C, with values remaining in the order of 10^8^ PFU/mL. In contrast, infectivity declined progressively at higher temperatures. A noticeable reduction was observed from 40 °C onward, with a more pronounced decrease at 50 °C and above. By 60 °C, phage titers had dropped by several orders of magnitude, and infective particles were nearly undetectable at 70 °C. These results indicate that AUBFM01 is stable under refrigerated, ambient, and physiological temperature conditions, but is sensitive to elevated heat.

The pH stability profile showed that AUBFM01 was best preserved under near-neutral conditions (Figure 5b). The highest infectivity was observed around pH 7, where titers remained close to 10^8^ PFU/mL. The phage also retained viability under mildly acidic to mildly alkaline conditions, particularly between pH 5 and 9, although titers were lower than those observed at neutrality. In contrast, infectivity was markedly reduced at more extreme pH values. Strongly acidic conditions and strongly alkaline conditions were associated with substantial losses in viable phage particles, with titers decreasing by multiple orders of magnitude. Collectively, these findings show that AUBFM01 is most stable under moderate temperature and near-neutral pH conditions, supporting its suitability for standard laboratory handling and further biological evaluation.

### 3.4. Biofilm Disruption

**Figure 6:**
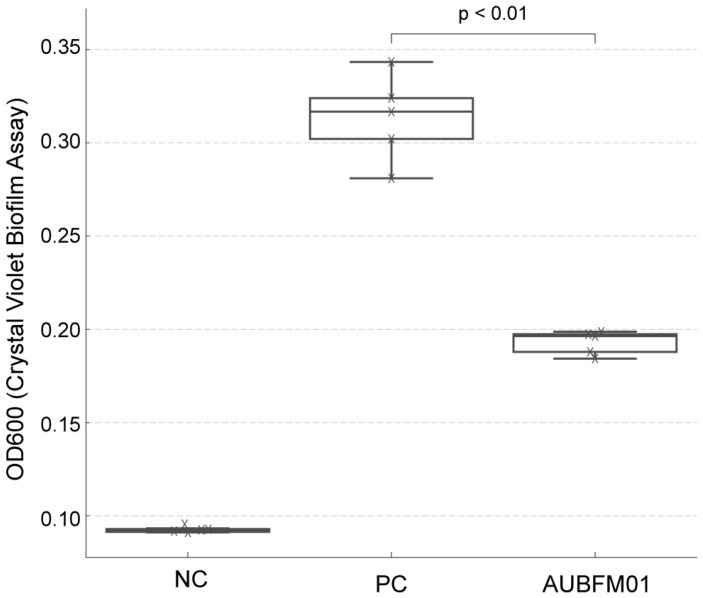
Antibiofilm activity of phage AUBFM01 against 24 h preformed *A. baumannii* biofilms, assessed by crystal violet staining. Compared with the untreated positive control (PC), AUBFM01 significantly reduced biofilm biomass, as reflected by lower OD600 values, although biomass remained above the negative control (NC) background level. The reduction in biofilm biomass in the AUBFM01-treated group was statistically significant (*p* < 0.01, *n* = 5 replicate wells).

The antibiofilm activity of AUBFM01 was evaluated against 24 h preformed *A. baumannii* biofilms using a crystal violet staining assay (Figure 6). As expected, the untreated biofilm control showed the highest biomass, with OD values of approximately 0.30–0.32, whereas the negative control remained near background levels (approximately 0.09). Treatment with AUBFM01 reduced biofilm biomass relative to the untreated control, with OD values decreasing to approximately 0.19–0.20. This corresponded to an estimated reduction of about 35–40% in biofilm biomass. The difference between the untreated control and the phage-treated group was statistically significant (*p* < 0.01), indicating that AUBFM01 was able to reduce established *A. baumannii* biofilms under the tested conditions. Although biofilm biomass was not reduced to background levels, the observed decrease suggests that AUBFM01 can partially disrupt mature biofilm structures in vitro.

### 3.5. Genomic Features and Phylogenetic Classification

**Figure 7:**
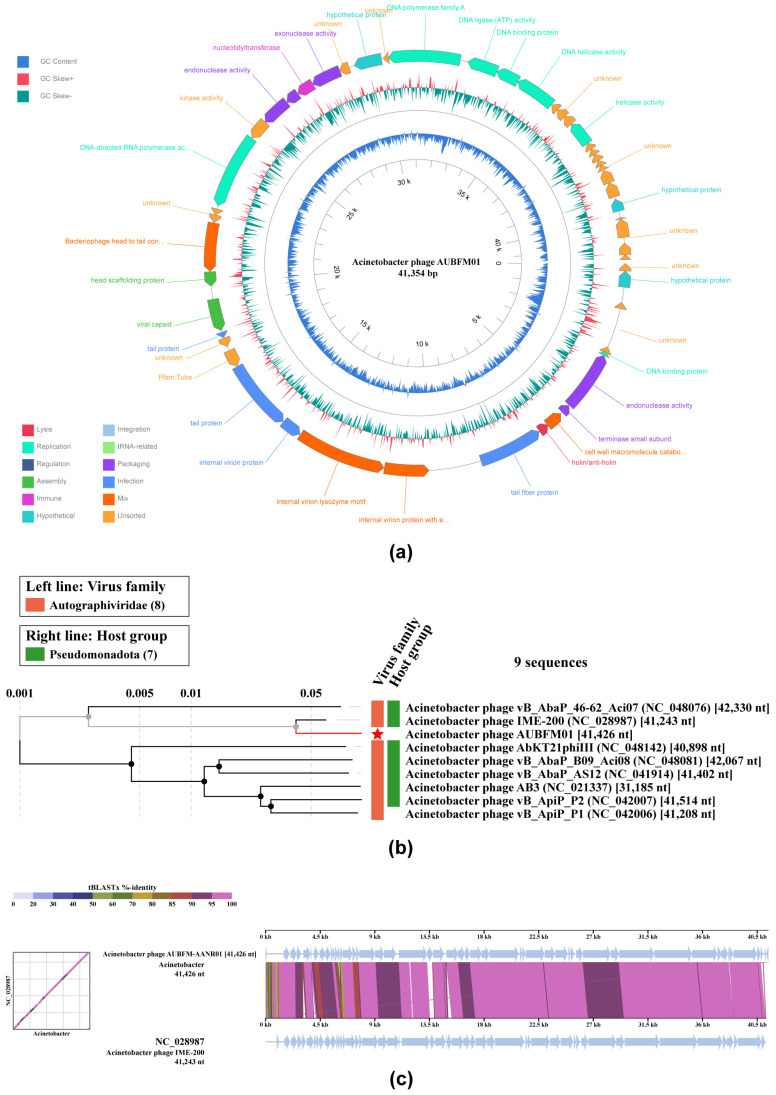
Genomic and phylogenomic characterization of phage AUBFM01. (**a**) Circular genome map of AUBFM01 showing the 41,354 bp dsDNA genome, predicted open reading frames, functional annotation, and GC content/GC skew profiles. Genes are organized into modules associated with replication, structural and assembly functions, DNA packaging, and host lysis. (**b**) Proteomic phylogenetic analysis placing AUBFM01 within the family *Autographiviridae*, clustering most closely with Acinetobacter phage IME-200 and related Friunavirus-like phages. (**c**) Whole-genome comparison between AUBFM01 and its closest relative, Acinetobacter phage IME-200 (NC_028987), showing high nucleotide similarity and extensive synteny across most of the genome, consistent with a closely related but distinct lytic phage.

Whole-genome sequencing showed that AUBFM01 is a double-stranded DNA phage with a genome size of 41,354 bp (Figure 7a). Genome annotation revealed a modular organization, with predicted open reading frames assigned to functional categories including DNA replication and metabolism, structural and morphogenesis proteins, DNA packaging, and host lysis. As shown in the circular genome map (Figure 7a), genes related to virion structure and assembly occupied a substantial portion of the genome, whereas replication- and lysis-associated genes were organized in distinct regions. This overall architecture is consistent with that commonly observed in lytic phages of the family *Autographiviridae*. No lysogeny-associated genes, including integrases or recombinases, were identified, and no known antibiotic resistance genes or bacterial virulence determinants were detected.

Phylogenomic analysis placed AUBFM01 within the family *Autographiviridae* and clustered it with Acinetobacter phages related to the *Friunavirus* group (Figure 7b). In the proteomic tree, AUBFM01 grouped most closely with Acinetobacter phage IME-200, indicating a close evolutionary relationship with previously described lytic Acinetobacter phages. Comparative genome alignment further supported this placement (Figure 7c). Whole-genome comparison between AUBFM01 and its closest reference sequence revealed a high degree of nucleotide conservation and extensive synteny across most of the genome, with only limited regions of divergence. The strong diagonal similarity pattern and the largely colinear genome blocks indicated broad conservation of gene order and overall genome architecture. Consistent with these observations, VIRIDIC analysis identified Acinetobacter phage IME-200 (NC_028987.2) as the closest sequenced relative of AUBFM01, with an intergenomic similarity of 91.8%, whereas the remaining related genomes showed lower similarity values, generally ranging from approximately 81.0% to 87.1% (Supplementary Figure S1). The aligned genome fraction and genome length ratio were both close to 1.0, indicating that these genomes are highly comparable in size and align across nearly their full lengths. Collectively, these findings classify AUBFM01 as a closely related but distinct Acinetobacter phage within the family *Autographiviridae*, with genomic features consistent with a lytic lifestyle.

### 3.6. Impact of AUBFM01 on THP-1 Macrophage Viability and Phenotype

**Figure 8:**
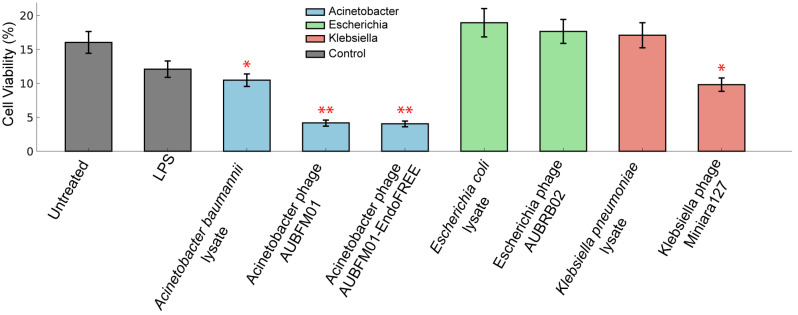
Effect of bacterial lysates and phage preparations on the viability of PMA-differentiated THP-1 macrophages after 24 h of exposure. Viability was assessed in untreated cells, LPS-treated cells, cells exposed to bacterial lysates, and cells treated with purified phage preparations from *Acinetobacter*, *Escherichia*, and *Klebsiella*. AUBFM01 and endotoxin-reduced AUBFM01 were associated with the greatest reduction in macrophage viability, whereas AUBRB02 showed minimal effect and Miniara127 produced an intermediate decrease. Bars represent mean viability ± SD from *n* = 3 independent biological replicates, and points represent individual biological replicates. Statistical significance was assessed by one-way ANOVA followed by Dunnett’s multiple-comparison test, with each condition compared against the untreated control. Significance levels are indicated as follows: * *p* < 0.05; ** *p* < 0.01.

The effect of purified phage preparations on PMA-differentiated THP-1 macrophages was first evaluated by viability analysis after 24 h of exposure (Figure 8).

Untreated cells showed the highest viability, at approximately 17–18%, whereas LPS treatment was associated with a moderate reduction to about 13–14%. Exposure to *A. baumannii* lysate alone further decreased viability to approximately 11–12%. The most pronounced reduction was observed in macrophages exposed to AUBFM01-associated conditions, in which viability decreased to approximately 4–5%. A comparable reduction was observed with both endotoxin-reduced AUBFM01 and non-endotoxin-reduced AUBFM01, suggesting that the effect was not solely attributable to residual endotoxin. Notably, this pattern was not observed with all phage preparations tested. *Escherichia* phage AUBRB02 and *E. coli* lysate had minimal effect on macrophage viability, with values remaining close to untreated levels, whereas *Klebsiella* phage Miniara127 was associated with an intermediate reduction in viability. Together, these findings indicate that AUBFM01 was associated with a distinct and substantially greater reduction in THP-1 macrophage viability than the other phage preparations examined.

**Figure 9:**
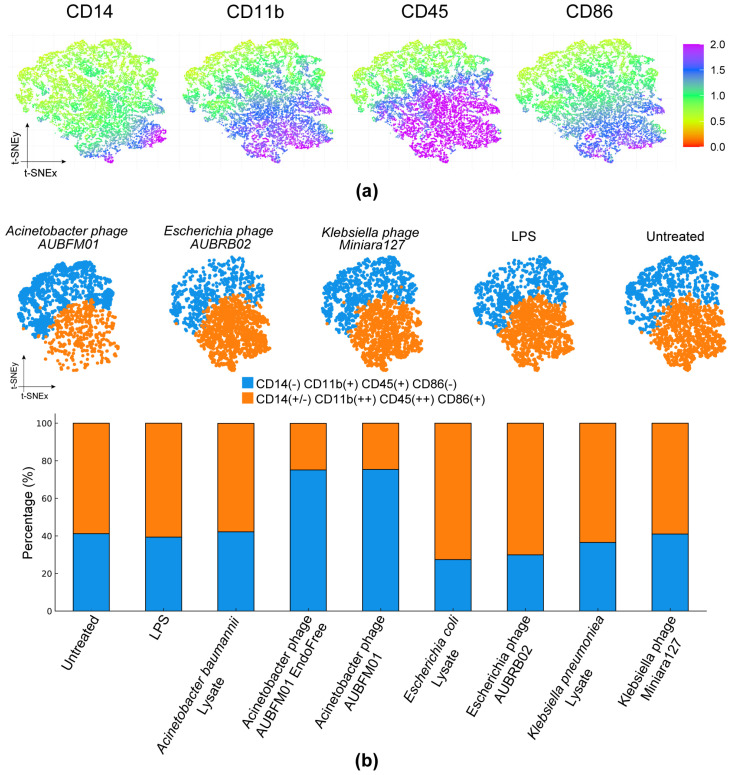
Phenotypic profiling of PMA-differentiated THP-1 macrophages after exposure to bacterial lysates, phage preparations, LPS, or no treatment. (**a**) t-SNE marker distribution maps based on CD14, CD11b, CD45, and CD86 expression showing the major phenotypic structure of the viable cell population. (**b**) K-means clustering identified two principal macrophage subsets: a CD14(-)CD11b(+)CD45(+)CD86(-) resting/intermediate-like population and a CD14(+/-)CD11b(++)CD45(++)CD86(+) activated-like population. Quantification of these subsets showed enrichment of the resting/intermediate-like population in AUBFM01- and endotoxin-reduced AUBFM01-treated cells, whereas untreated, LPS-treated, and most other phage- or lysate-exposed conditions remained enriched in the activated-like population.

To assess whether AUBFM01 exposure was also associated with phenotypic changes in the surviving macrophage population, flow cytometry data were analyzed using t-SNE based on CD14, CD11b, CD45, and CD86 expression (Figure 9a), followed by k-means clustering to identify major cell groupings (Figure 9b). The marker distribution maps indicated that CD45 and CD86 contributed most strongly to the separation of cell states, while CD14 and CD11b further refined the phenotypic distribution across the embedding. Based on the combined marker-expression patterns, two principal populations were identified: a CD14(-/+)CD11b(+)CD45(++)CD86(+) population, here referred to as an activated-like phenotype, and a CD14(-)CD11b(-/+)CD45(+)CD86(-) population, corresponding to a resting/intermediate-like phenotype. Quantification of these populations revealed clear condition-dependent differences (Figure 9b). Untreated cells, LPS-treated cells, and cells exposed to *A. baumannii* lysate alone were enriched in the activated-like population, which represented approximately 60–65% of the total cells. In contrast, exposure to AUBFM01 was associated with enrichment of the resting/intermediate-like population. In the endotoxin-reduced AUBFM01 condition, approximately 75–80% of cells were assigned to the resting/intermediate-like subset, whereas a similar distribution was observed with AUBFM01, where this population represented approximately 70–75% of the remaining cells. This pattern differed from the *Escherichia* phage-associated conditions, which remained enriched in activated-like cells, and from the *Klebsiella* phage condition, which showed a more balanced distribution but did not reproduce the marked shift observed with AUBFM01.

Overall, these results indicate that, under the tested conditions, AUBFM01 exposure was associated with both a marked reduction in THP-1 macrophage viability and an altered phenotypic distribution among the surviving cells, characterized by enrichment of a resting/intermediate-like population. This pattern distinguished AUBFM01 from the other phage preparations included in the analysis. However, because the phenotypic assessment was performed on the residual viable population, these findings should be interpreted cautiously and do not by themselves distinguish between true phenotypic reprogramming and selective survival of specific cell subsets.

## 4. Discussion

The increasing prevalence of multidrug-resistant *Acinetobacter baumannii* has reduced the effectiveness of conventional antibiotics and reinforced the need for alternative antibacterial strategies [5]. In this study, we isolated and characterized AUBFM01, a lytic phage belonging to the family *Autographiviridae* [24], and performed an initial assessment of its interaction with human macrophage-like cells [12,25]. Overall, our findings indicate that AUBFM01 possesses antibacterial activity against *A. baumannii*, including dose-dependent lytic activity in liquid culture [26,27] and partial disruption of preformed biofilms [28,29], and is also associated with distinct effects on THP-1-derived macrophages [14]. These results support the importance of evaluating both antibacterial performance and host–cell interactions during early phage characterization [3,30].

From an antibacterial perspective, AUBFM01 showed relatively rapid adsorption, a short latent period, and measurable suppression of *A. baumannii* growth, properties generally considered favorable for lytic phages intended for antibacterial use [31,32]. AUBFM01 also reduced the biomass of preformed *A. baumannii* biofilms in vitro, which is relevant given the role of biofilms in persistence, device colonization, and increased tolerance to antimicrobial treatment and host defenses [33]. In addition, the translucent halos observed around plaques may be consistent with depolymerase-associated activity, as previously described for some *A. baumannii* phages [33]. However, this interpretation remains tentative in the absence of direct functional or genomic confirmation.

The genome of AUBFM01 further supports its interest as an anti-*Acinetobacter* phage. No lysogeny-associated genes, antibiotic resistance determinants, or known bacterial virulence factors were identified, consistent with the profile expected for a lytic phage being considered for antibacterial applications [34]. In addition, AUBFM01 remained stable under moderate temperature conditions and near-neutral pH, supporting its suitability for standard laboratory handling and further preclinical evaluation [31,32].

The most distinctive finding of this study was the macrophage-associated profile of AUBFM01. Flow cytometry-based phenotypic analysis showed that LPS- and bacterial lysate-treated macrophages were enriched in a CD86-positive activated-like population, whereas AUBFM01 exposure was associated with enrichment of a resting/intermediate-like population among the remaining viable cells [35,36]. A similar pattern was observed after endotoxin reduction, suggesting that the effect was not solely explained by residual endotoxin contamination. This is broadly consistent with the growing literature indicating that bacteriophages are not necessarily immunologically inert and may interact with macrophages in diverse, phage-dependent ways [37]. At the same time, the present data do not establish the mechanism underlying this effect, and direct involvement of specific uptake pathways or signaling receptors remains speculative [38].

An additional consideration is that untreated PMA-differentiated THP-1 macrophages displayed a phenotypic profile broadly similar to that of LPS-treated cells. This may reflect the tendency of PMA-differentiated THP-1 cells to retain a partially activated basal state even after a resting period [39,40], which can reduce the apparent separation between untreated and stimulated conditions when a limited marker panel is used [41]. This model-related feature should therefore be taken into account when interpreting the clustering results [42].

A key point in interpreting the immune data is that AUBFM01 also produced the largest reduction in THP-1 viability among the tested conditions. Therefore, enrichment of a resting/intermediate-like population among surviving cells may reflect true phenotypic modulation, selective survival of specific subsets, or a combination of both. For this reason, the present findings should be interpreted as evidence of a distinct macrophage-associated effect rather than definitive proof of direct immunosuppressive reprogramming [9]. Nevertheless, the fact that AUBFM01 differed from the other phage preparations tested supports the view that phage–macrophage interactions should be assessed on a case-by-case basis [11].

Several limitations should be acknowledged. The immune analyses were performed only in PMA-differentiated THP-1 macrophages, and the phenotypic assessment relied on a restricted marker panel. In addition, no cytokine, chemokine, or transcriptional analyses were performed, and the marked reduction in viability limits interpretation of the phenotypic shift. Although endotoxin depletion reduced the likelihood that the observed effect was driven solely by endotoxin contamination, further controls will be needed to determine whether the response is attributable to intact phage particles, phage-associated components, or other preparation-specific factors. Finally, all findings were obtained under in vitro conditions, and their relevance to in vivo efficacy, phage clearance, and therapeutic performance remains to be established.

Overall, AUBFM01 emerges as a lytic anti-Acinetobacter phage with measurable antibiofilm activity and a distinct macrophage-associated profile that warrants further mechanistic and translational investigation. Future studies should include cytokine profiling, broader macrophage phenotyping, cell-death analyses, validation in primary macrophages, and infection-relevant in vivo models to better define the biological significance of the effects observed here.

## 5. Conclusions

AUBFM01 is a lytic *Acinetobacter baumannii* phage with favorable in vitro antibacterial characteristics, including efficient host adsorption, rapid replication, and measurable antibiofilm activity, together with a genome consistent with a lytic lifestyle and lacking detectable lysogeny-associated genes, known virulence factors, or antibiotic resistance determinants. In parallel, its association with altered macrophage viability and phenotype highlights that early phage characterization may benefit from going beyond antibacterial performance alone. Rather than establishing a definitive immune profile, the present study provides an initial immune-aware framework for evaluating anti-Acinetobacter phages. Overall, these findings support AUBFM01 as a promising candidate for further investigation and emphasize the value of integrating antibacterial, genomic, and host-response assessments in the preclinical evaluation of therapeutic phages.

## Data Availability

The complete genome sequence of Acinetobacter bacteriophage AUBFM01 has been deposited in GenBank under accession number PX094877 (supplementary information AUBFM01.gbk). The raw sequencing reads are available in the Sequence Read Archive (SRA) under BioProject ID PRJNA613441. Associated biosample information for the bacterial strains used in this study can be accessed via the following Biosample accession numbers: SAMN38010243, SAMN38010259, SAMN38010261, SAMN38010265, SAMN38010269, SAMN38010271, SAMN38010273. All other relevant data supporting the findings of this study are included in the article/Supplementary Material. Further inquiries can be directed to the corresponding author.

## Supplementary Materials

The following supporting information can be downloaded at: https://www.mdpi.com/article/10.3390/microorganisms14040903/s1, Figure S1: Intergenomic similarity analysis of AUBFM01 and related *Autographiviridae* bacteriophages. Heatmap showing pairwise intergenomic similarity between the genome of AUBFM01 (NODE_1_length_41354_cov_1450.672375) and closely related reference bacteriophage genomes retrieved from GenBank. The percentage values within the matrix indicate intergenomic similarity for each genome pair. The bars above the heatmap represent genome length, while the lower triangle annotations indicate aligned genome fraction and genome length ratio. The analysis shows that AUBFM01 shares high genomic relatedness with the compared phages, with similarity values ranging approximately from 81% to 92%, supporting its close relationship to members of this phage group.

## Abbreviations

The following abbreviations are used in this manuscript:

ATCC	American Type Culture Collection
CFU	colony-forming unit
CO_2_	carbon dioxide
dsDNA	double-stranded DNA
EU	endotoxin units
FBS	fetal bovine serum
FCS	flow cytometry standard
LB	Luria–Bertani
LPS	lipopolysaccharide
MDR	multidrug-resistant
MOI	multiplicity of infection
NC	negative control
OD	optical density
PBS	phosphate-buffered saline
PC	positive control
PEG	polyethylene glycol
PFU	plaque-forming unit
PMA	phorbol 12-myristate 13-acetate
RPMI	Roswell Park Memorial Institute medium
SD	standard deviation
SM	saline magnesium
SRA	Sequence Read Archive
THP-1	human monocytic leukemia cell line
t-SNE	t-distributed stochastic neighbor embedding
