# Supplementary figures and images for: AUBFM01 Phage as a Therapeutic Candidate Against MDR Acinetobacter baumannii: Characterization, and Immune-Aware Profiling
Source: Microorganisms. 2026 Apr 16;14(4):903. doi: 10.3390/microorganisms14040903 (PMC13119066; doi:10.3390/microorganisms14040903)

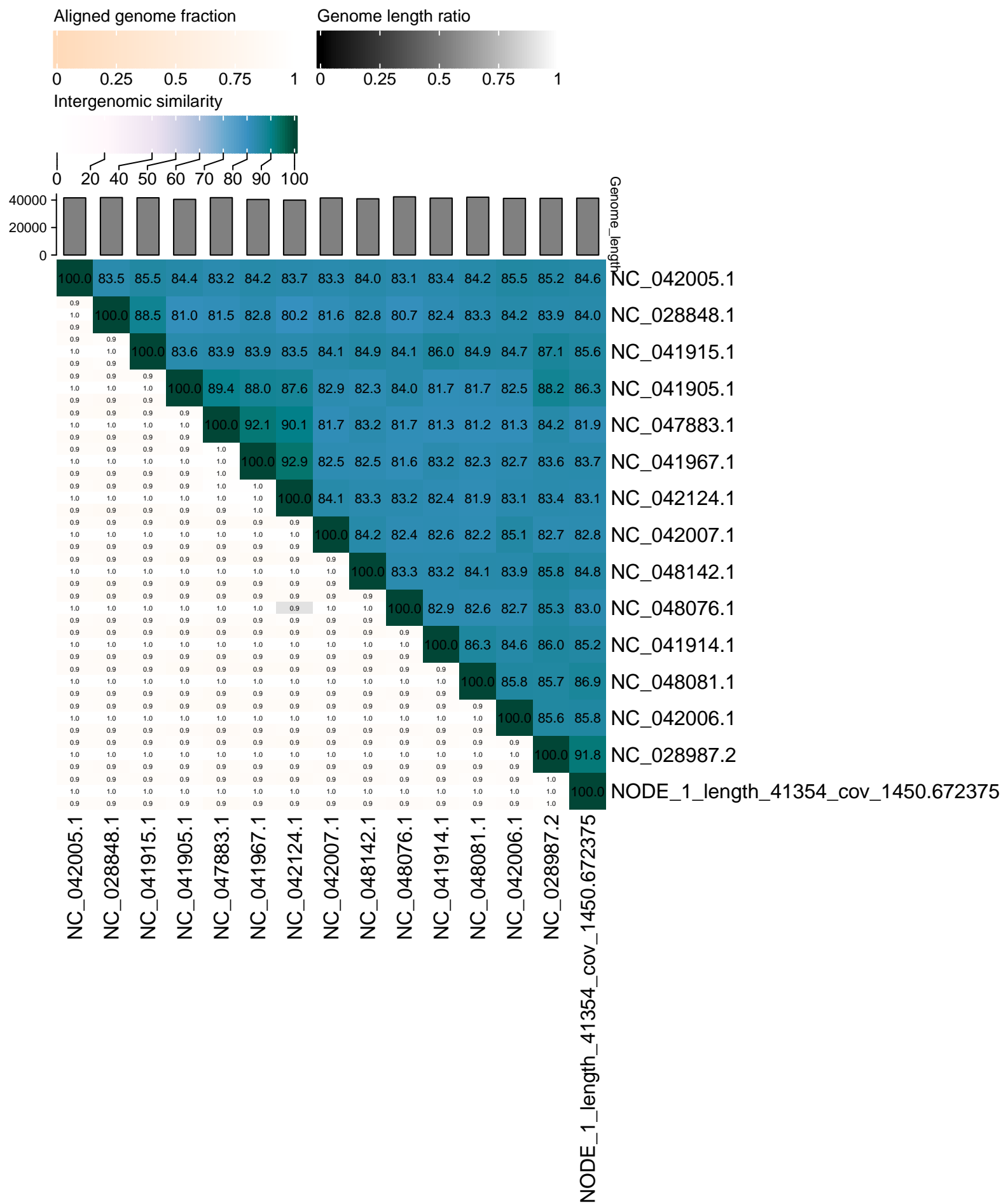

Supplement: Supplementary file 1 [file microorganisms-14-00903-s001.zip › Supplementary Files/Supplementary Figure S1.pdf]
